# Noncompetitive Inhibition of Bovine Liver Catalase by Lawsone: Kinetics, Binding Mechanism and *in silico* Modeling Approaches

**DOI:** 10.22037/ijpr.2019.111600.13255

**Published:** 2020

**Authors:** Simin khataee, Gholamreza Dehghan, Samaneh Rashtbari, Siavash Dastmalchi, Mehrdad Iranshahi

**Affiliations:** a *Department of Biology, Faculty of Natural Sciences, University of Tabriz, Tabriz, Iran.*; b *Biotechnology Research Center, School of Pharmacy, Tabriz University of Medical Sciences, Tabriz, Iran.*; c *Faculty of Pharmacy, Near East University, POBOX: 99138, Nicosia, North Cyprus, Mersin 10, Turkey. *; d *Biotechnology Research Center, Pharmaceutical Technology Institute, Mashhad University of Medical Sciences, Mashhad, Iran.*

**Keywords:** Bovine liver catalase, Lawsone, Molecular docking, Naphthoquinones, Noncompetitive inhibition

## Abstract

Lawsone (2-hydroxy-1,4-naphtoquinone; LAW), as a naphthoquinone derivative, is the biologically active component of *Henna* leaves. In this study, the structural and functional effects of LAW on bovine liver catalase (BLC), has been studied utilizing ultraviolet-visible (UV-vis) absorption, fluorescence, and ATR-FTIR spectroscopic techniques, and molecular docking approach. *In-vitro* kinetic study showed that by adding gradual concentrations of LAW, catalase activity was significantly decreased through noncompetitive inhibition mechanism. UV–vis and ATR-FTIR spectroscopic results illustrated that additional concentration of LAW lead to significant change in secondary structure of the enzyme.The fluorescence spectroscopic results at different temperatures indicated that LAW quenches the intrinsic fluorescence of BLC by dynamic mechanismand there is just one binding site for LAW on BCL. Changing the micro-environment nearby two aromatic residues (tryptophan (Trp) and tyrosine (Tyr)) were resulted from synchronous fluorescence. The thermodynamic parameters were implied that the hydrophobic bindings have a significant impress in the organization of the LAW-catalase complex. Molecular docking data in agreement with experimental results, confirmed that hydrophobic interactions are dominant. Inhibition of enzyme activity by LAW, showed that along withits helpful effects as ananti-oxidant compounds, the side effects of LAW should not be overlooked.

## Introduction

Catalase (H_2_O_2 _oxidoreductase, EC 1.11.1.6; CAT)(PDB ID 1TGU), is an antioxi-dative and protective enzyme that plays critical role in organelles and tissues to detoxify the impacts of hydrogen peroxide (H_2_O_2_)(1, 2). It is a ubiquitous enzyme of almost all aerobic organisms and many anaerobic organ-isms (3). Catalase deficiency leads to the steady state concentration of H_2_O_2_, which may raise various pathological conditions as cardiovascular disease, cancer, ageing oxidative, hypertension, Alzheimer, diabetes mellitus,andacatalasemia (4, 5).

Catalase from Bovine liver (BLC) (MW = 240000 Da) is a homotetramericheme-containing enzyme, with four equal-size subunits. Each subunit (MW = 60 kDa) of this enzyme organized of 253 pairs of amino acids, one NADPH as a cofactor (per subunit) and heme prosthetic that are strongly connected to the structure of CAT through tyrosine, histidine, and asparagine (Y415, H75 and N201) as ligand(6).

Also each subunit divides into four domains: a conserved eight-stranded β-barrel which generate the hydrophobic core per subunit, wrapping loop which attaches the β-barrel to N-terminal threading arm, C-terminal helices which surround the β-barrel, and N-terminal threading arm(7). Catalase structure has central cavity and three access channels that convert substrate to the active site.The channels provide substrate access to active site and allow release of product(8).

BLC, can strongly bind to NADPH (as cofactor (in the middle of the β-barrel and the helical domain (9).The function of the NADPH is reducing the susceptibility of enzyme to prevent inactivation of CAT at low concentrations of its substrate (10).NADPH plays the role of an electron donor by transformation of compound II to restore the active form of the catalase (11). In accordance with BLAST (Basic Local Alignment Search Tool) consequences, bovine liver catalase includes efficient similarities with human catalase (12).So, BLC can be utilized as a model enzyme of human liver CAT.

**Figure 1:**
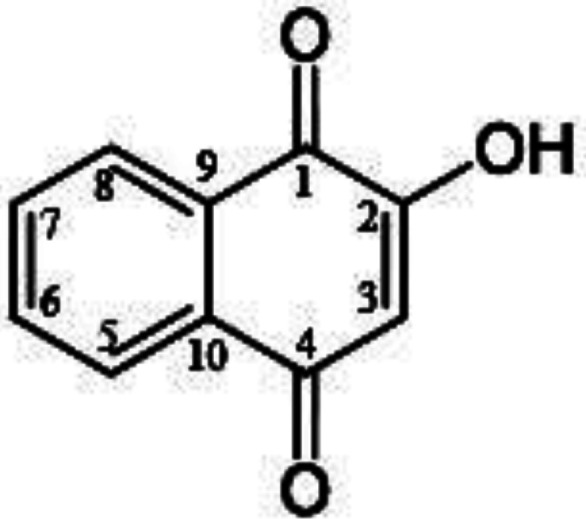
Chemical structure of lawsone (2-hydroxy-1,4-naphtoquinone).

Naphthoquinones are ubiquitous in nature and they have been seem to have a lot of biological activities and medicinal properties, such as cytotoxic, anti-oxidant, anti-cancer, anti- fungal, anti-bacterial, and anti-tumoral effects,(13)which can play a main role in the human defense system (14).1,4-Naphthoquinones, as anti-cancer compound are found in a number of aminoquinones such as actinomycins, streptonigrin, etc (14, 15). An important derivative of 1,4-naphthoquinone is lawsone (LAW). LAW (2-hydroxy-1,4-naphtoquinone), the active component of Henna leaves (*Lawsonia inermis* Linn), has a various kind of biological activities such as antioxidant, antibacterial, antifungal, anti-inflammatory, antipyretic, and anticancer. It is a red-orange pigment and partially water-soluble (14, 16).The chemical formula of LAW is C_10_H_6_O_3_ corresponding to the total molecular weight of 174.16 atomic mass units. In the chemical structure of LAW, two oxygen atoms are connected to the carbons of naphthalenic at positions of 1 and 4 for generation of 1, 4-naphthoquinone. A hydroxyl group (–OH) of enolic temper is present at the position 2 (Figure 1)(16, 17).

Reactive oxygen species (ROS), extremely reactive molecules, exist in elevated levels in cancer cells because of their high metabolic activity and play a main role in carcinogenesis through induction of DNA damage. Cells detoxify ROSs and maintain intracellular homeostasis by using different antioxidant enzymes including catalase, glutathione peroxidase and superoxide dismutase (SOD) (18). Decreased antioxidant enzymes activity can be associated with different types of cancer (19). Literatures support anticancer potential of napthoquinone derivatives such as LAW in various types of cancer. However, it has been reported that the increase in ROS can induce tumor cell death through damaging function of ROS and induction of apoptosis in tumor cells through death signaling pathways. So, studying effects of different compounds on the activity of antioxidant enzymes and induction of oxidative stress by increasing ROS, can be one of the main and interesting issues in cancer therapy. In the present study the impact of LAW on the function and structure of CAT, as a most important antioxidant enzyme, was studied *in-vitro* using multiple spectroscopic methods and molecular docking technique and its therapeutic potential was investigated in treatment of cancer by decreasing CAT activity. 

## Experimental

*Materials*

Catalase from bovine liver and dimethyls-ulfoxide (DMSO) were purchased from Sigma Chemical Company (St Louis, Missouri).LAW was obtained from Prof. Iranshahi’s laboratory, Mashad University of Medical Science. Sodium phosphatesalts and hydrogen peroxide (H_2_O_2_) 30% were acquired from Merck Co (Darmstadt, Germany). The LAW stock solution was freshly prepared by dissolving it in DMSO and was mixed with sufficient phosphate buffer to obtain the desired volume. The concentration of H_2_O_2_ stock solution was accounted spectrophotometrically through the molar extinction coefficient of 40 M^−1^cm^−1^.

*Methods*

*Kinetics studies*

Catalase (4 nM) activity was assessed by following the decline in themaximum absorbance of H_2_O_2_ at 240 nm and at 25 °C, based on its decomposition by the enzyme using a UV spectrophotometer (T-60, PG Instrument UK)(3).The assays were accomplished in 3 mLcuvette,including50 mM sodium phosphate buffer (pH 7), different concentrations of H_2_O_2_ (10-70 mM) and 10 μL of CAT suspension (11).In order to study the structural and functional alterations of enzyme by LAW, CAT into the reaction medium was incubated with different concentrations of LAW (1.42, 2.84, 5.68, 11.36 and 17.04μM) for 3 minutes. Subsequently, an appropriated concentration of substrate (55.5 mM) was added to the mixture and the alteration in the absorbance was monitored in A_240_. 

*Fluorescence measurements*

In order to assess the three-dimensional conformational changes of CAT through increasing concentrations of LAW (0.47, 4.7, 11.8, 14.2, 23.6 and 37.8µM), fluorescence intensity evaluations were recorded utilizing a FP-750 Jasco spectrofluorometer (Kyoto, Japan) with 1 cm quartz cells(20). The excitation wavelengths (λ_ex_) fitted at 280 nm and the fluorescence spectra were recorded from 300 to 500 nm (λ_em_) at two temperatures (25, 30 and 37 °C). 

Synchronous fluorescence spectra of BLC were evaluated using Δλ=15 nm (λ_em_=270-330) and Δλ=60 nm (λ_em_=310-380) to obtain the fluorescence spectra of tyrosine (Tyr) and tryptophan (Trp) residues, respectively. The slit widths and the scan speed of both emission and excitation were set at 10 nm and1000 nm min^−1^, respectively.

*UV–vis and ATR-FTIR spectroscopies*

The UV-vis spectrophotometer (T-60, PG Instrument UK) was utilized to assess the UV-vis absorption spectra of CAT in the spectral range of 200-500 nm, without and with gradual concentrations of LAW.

To elucidate the protein-ligand binding feature, Fourier transform infrared spectro-scopy (FT-IR) was carried out with a Bruker, Tensor 27 FT-IR spectrometer equipped with a KBr and ZnSe (beam splitter), Attenuated Total Reflection (ATR) technique with resolution of 1cm^-1 ^and DLa TGA detector. The FT-IR spectrum of native CAT was obtained in sodium phosphate buffer, at 1200 cm^-1^to 1800 cm^-1 ^and compared with the results of CAT-LAW complex spectra (9.4 and 23.6 µM of LAW).

*Molecular docking investigation*

The molecular docking analysis was done to detect the best binding site of CAT for LAW using AutoDock 4.2 software. The Protein Data Bank (RCSB) was used to take crystallography structure of CAT. The 3Dstructure of LAW was drawn by Hyperchem 8.0.6 and the minimizing of the energy was performed by Gaussian 98 program (21-23).The Lamarckian genetics algorithm technique was used to search connection site of LAW on CAT. Finally, The Auto Dock Tools 1.5.4 package were used to provide the analyzed mode of binding between LAW and CAT (3).

## Results and Discussion

*Functional effects of LAW on CAT (kinetics studies)*

**Figure 2:**
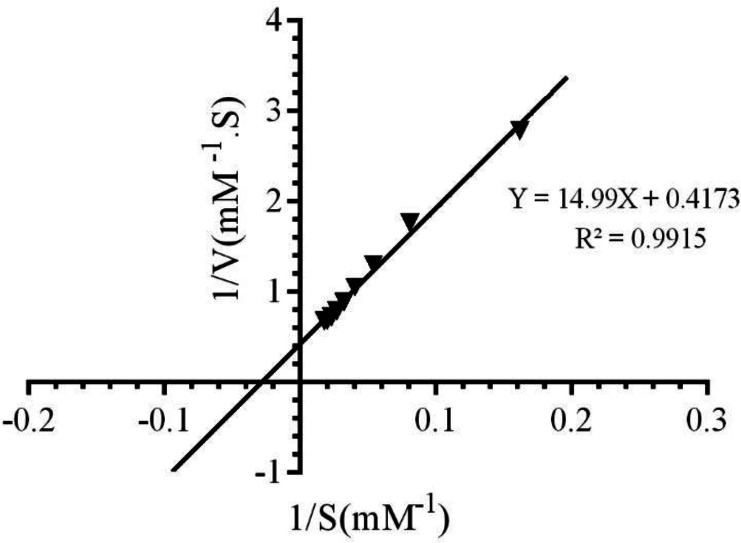
Lineweaver-Burk plot of bovine liver catalase activity in the presence of different concentrations of H2O2 (10‐70mM) in 50 mM phosphate buffer, pH 7.0 at 298 K

To study the impact of LAW on the function of CAT, the UV-vis spectrophotometer was applied. At first, the kinetic parameters of CAT were determined by assaying enzyme in the presence of incremental amounts of H_2_O_2_ (10-70 mM) and appropriate concentration of CAT (4 nM). The rate of decomposition of substrate by CAT was evaluated at 240 nm and reduction in H_2_O_2_ absorption per unit time that was considered as enzyme function at 25 °C. Based on our previous studies, BLC activity was increased with raising the substrate concentration from 10 to 55.5 mM. Addition of higher concentration of H_2_O_2_ (more than 55.5 mM) significantly reduced enzyme activity due to inhibitory effect of substrate(21). Different kinetic parameters of CAT have been calculated by Lineweaver-Burk plot, as it is shown in Figure 2 and equation 1.

$\frac{1}{V_{\max}}=\frac{k_{m}}{V_{\max}}.\frac{1}{\left[S\right]}+\frac{1}{V_{\max}}$                    (1)

The values of K_m_, Michaelis-Menthen constant, and *V*_max_ for the free enzyme were obtained as 35.92 mM and 2.39 mM.S^-1^, respectively. Also, the value of *K*_cat_, catalytic constant was determined as 5.9 × 10^5^ s^-1^, using equation (2): 

$V_{\max}=K_{\mathrm{cat}}.[E_{t}]$                    (2)

where, [Et] incorporates the total CAT concentration. 

**Figure 3:**
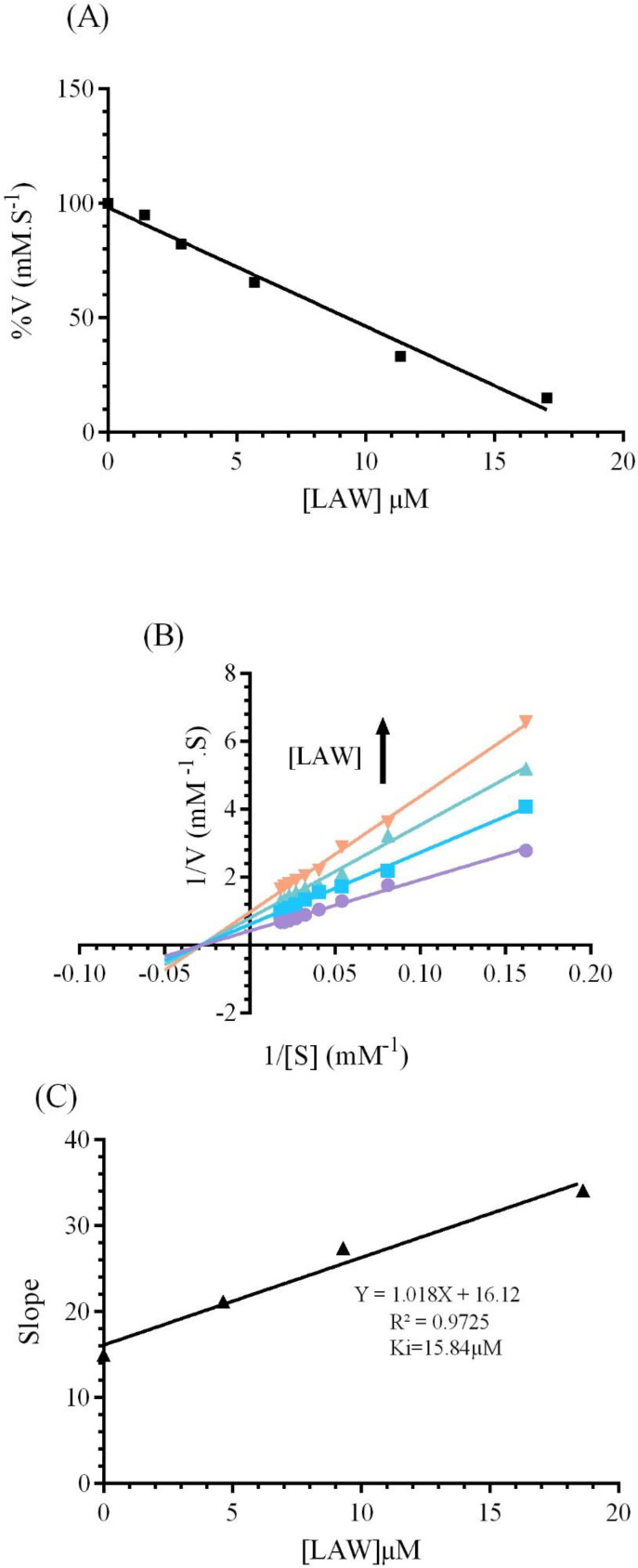
(A) The effect of various concentrations of LAW (0, 1.4, 2.84, 5.68, 11.36 and 17.04 µM) on the activity of BLC (4 nm) in 50 mM phosphate buffer, pH 7 at 298 k. (B) Lineweaver-Burk plot of BLC (4 nm) with and without various concentrations of LAW: 0, 4.6, 9.3, 18.6 µM in 50 mM phosphate buffer, pH 7, 298 k. (C) Secondary plot of BLC activity using the slopes of the primary Lineweaver–Burk plots versus concentrations of LAW

To identify the alterations in the function of CAT by LAW, the enzyme activity was evaluated in the presence of increasing concentrations of LAW (1.42, 2.84, 5.68, 11.36 and 17.04 μM) and optimum concentration of H_2_O_2_ (55.5 μM). The results of this experiment showed that an increase in the concentrations of LAW cause a significant decrease in the enzyme activity. The value of IC_50_, was approximated 9.3 μM using Figure 3A.

Three different types of reversible inhibitory mechanism for the enzyme have been defined: (a); competitive inhibition in which the inhibitor and free enzyme connect to each other through enzyme active site (b); noncompetitive inhibition in which inhibitor connects to both the free enzyme at a site that is different from the substrate binding site and the enzyme–substrate complex and (c); uncompetitive inhibition in which inhibitor can bind reversibly to the enzyme–substrate complex to yield anticompetitive inhibition (24).To realize the inhibitory mechanism of CAT by LAW, alterations in the enzyme activity were measured after forming the complex with different concentrations of LAW (4.6, 9.3 and 18.6 µM). The Lineweaver–Burk plots were drawn (Figure 3B). The kinetic parameters (*K*_m_ and *V*_m_) were computed using Lineweaver-Burk plots and compared with kinetic parameters of native enzyme(25). With increasing the concentration of LAW, the *K*_m_ (35.92) didn’t show significant alteration in the presence of LAW, whereas values of *V*_max_ of the enzyme decreased from 2.39 mM.s^-1^ for free BLC to 1.64mM.s^-1^ ,1.26mM.s^-1^and 1.036mM.s^-1^ for the enzyme in the presence of incremental concentration of LAW (4.6, 9.3 and 18.6 µM). As a result, LAW acts as a non-competitive inhibitor for catalase. The *K*_i _value was obtained from the equation (3) and (Figure 3c) as 15.77µM. 

$\frac{1}{V_{0}}=\frac{k_{m}}{V_{\max}}.\frac{1}{\left[S\right]}+\frac{1}{V_{\max}}.(1+\frac{\left[I\right]}{K_{i}})$                    (3)

*Fluorescence spectroscopy*

**Figure 4:**
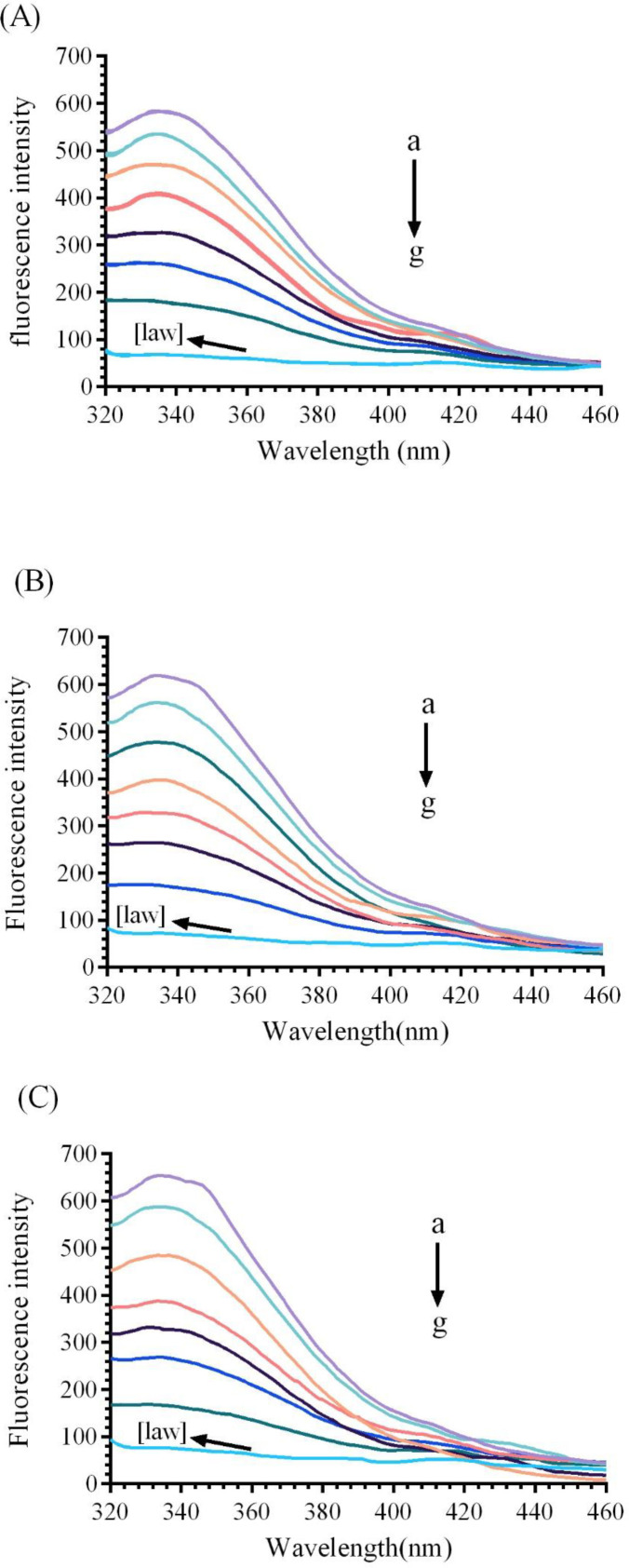
Fluorescence emission spectra of bovine liver catalase (BLC) in the presence or absence of different concentrations of LAW at 280 nm excitation wavelength, BLC concentration: (0.7 µM); LAW concentrations:(a) 0, (b) 0.47, (c) 4.7, (d) 11.8, (e) 14.2, (f) 23.6 and (g) 37.8 µM, (h) only LAW, 0.7 µM, (A) 298 K, (B) 303 K, (C) 310 K

The fluorescence spectroscopy was performed to predict the changes in the protein structure through interaction with various ligands. It gives valuable information around structural changes, binding mode, quenching mechanism, and binding constants (22).Fluorescence quenching is the reduction of fluorescence intensity of a fluorophore which results from some processes such as molecular rearrangements, energy transfer and etc (26). Bovine liver catalase consists of four polypeptide subunit, which each subunit contains 6tryptophan (Trp) and twenty Tyr residues (2, 11).Three aromatic residues (tyrosine, tryptophan, and phenyl alanine) of BCL lead to intrinsic fluorescence emission. In this experiment, the 280 nm as an excitation wavelength has been used to measure the fluorescence quenching mechanisms of CAT(21). Native enzyme shows a fluorescence emissions peak at 338 nm. As it can be observed in Figure 4, the fluorescence spectrum of the CAT gradually reduces with increasing the concentrations of LAW (0.47, 4.7, 11.8, 14.2, 23.6 and 37.8µM) at 25, 30, and 37 °C. 

**Figure 5:**
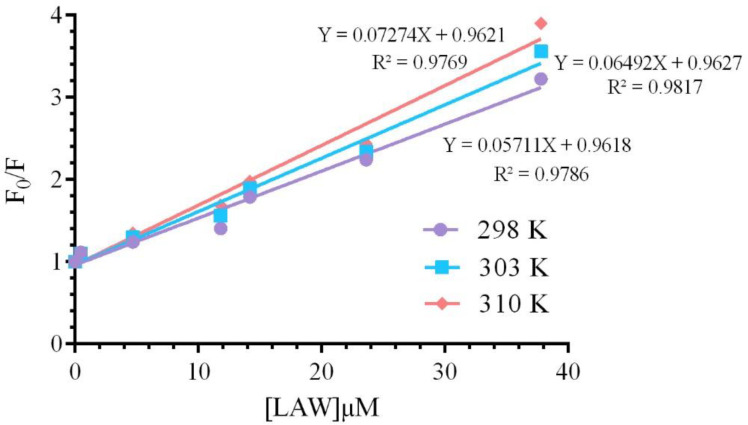
Stern- Volmer plots for the bovine liver catalase fluorescence quenching by LAW at 298, 303 and 310 K

Static and dynamic quenching, as two distinct quenching mechanisms, derive from the dependence of ligand-biomolecule interaction process on temperature. In static quenching, enhancing temperature leads to reduce the bimolecular quenching constant, but in dynamic quenching, enhancing temperature cause increase in the value of quenching constants (27). To confirm the quenching mechanism of CAT in the presence of LAW, Stern-Volmer equation (4) was used;

$\frac{F_{0}}{F}=1+K_{\mathrm{sv}}[Q]$                    (4)

where, *F* and *F*_0_ are the fluorescence intensities of enzyme with and without the quencher (LAW), respectively, *[Q]* is the total concentration of the quencher (LAW), *K*_SV_ is the Stern–Volmer quenching constant (28).The *K*_SV _value was calculated applying the *F*_0_*/F vs. [Q] *plot (Figure 5) at two different temperatures and are listed in Table 1. These data indicated that the values of *K*_SV_ increase with raising temperature, so the quenching mechanism is dynamic.

*Synchronous fluorescence study*

**Figure 6:**
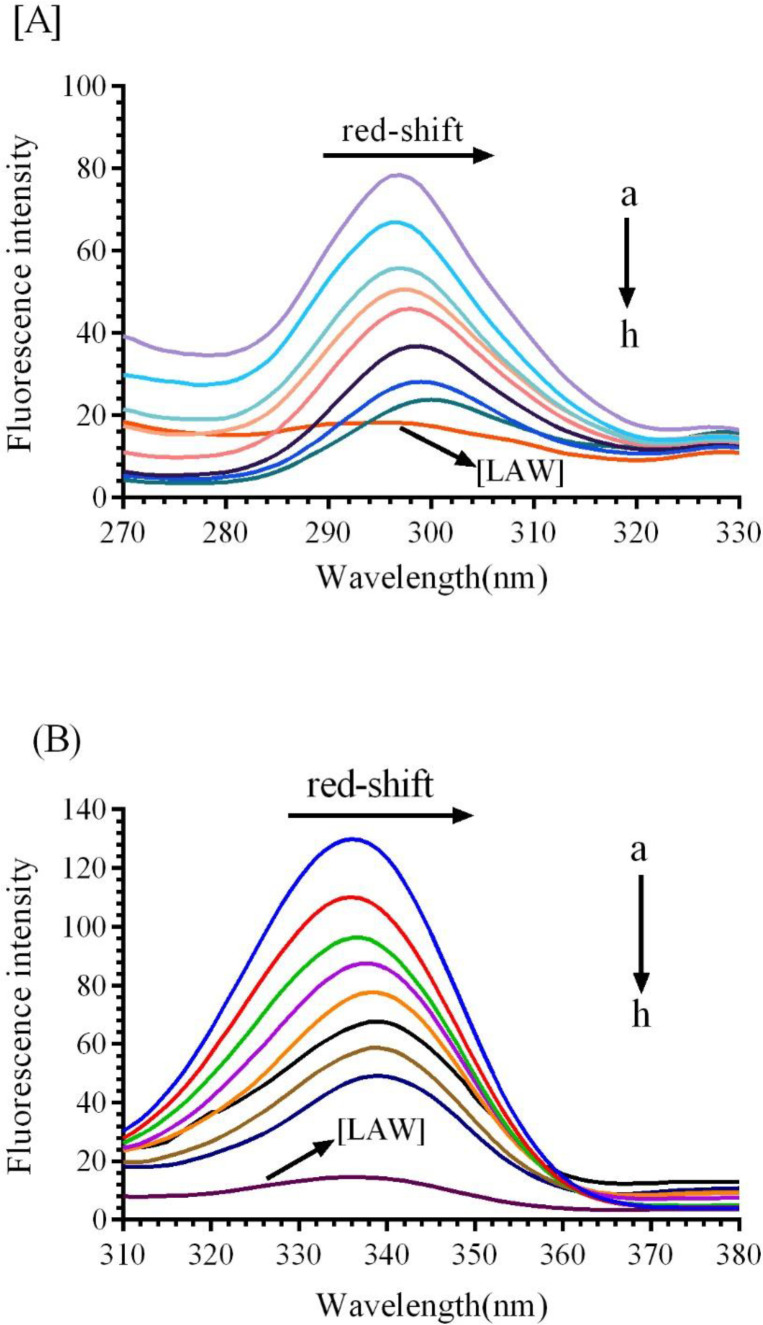
Synchronous fluorescence spectra of BLC in the presence of incremental concentration of LAW (a) 0, (b) 0.47, (c) 4.7, (d) 9.4, (e) 11.8, (f) 14.2, (g) 23.6 and (h) 37.8µM, (i) enzyme free LAW (0.7 µM); (A) ∆λ=15 and (B) ∆λ=60 nm at 298k

Synchronous fluorescence was applied to notify significant report about the microenvironment of CAT fluorophore groups (Trp and Tyr). In synchronous fluorescence, the spectra of molecules are taken from simultaneously scanning the excitation and emission spectra, when the wavelength intervals (∆λ) maintained at 15 nm and 60 nm for Tyr and Trp, respectively (29).Additional concentrations of LAW lead to gradual reduce in the fluorescence intensity of BLC indicating complex formation between BLC and LAW. As shown in Figure 6, different concentrations of LAW induce a clear red shift in ∆λ=60 nm and very small red shift in ∆λ=15 nm in comparison with the native CAT, implying that the Trp and Tyr are located in a polar environment.

*Thermodynamic parameters and binding studies*

The number of binding sites (*n*) and binding constant *(K*_a_*)*, were obtained from the equation 5;

$\log\frac{F_{0}-F}{F}=\log K+\mathrm{nlog}[Q]$                     (5)

**Figure 7:**
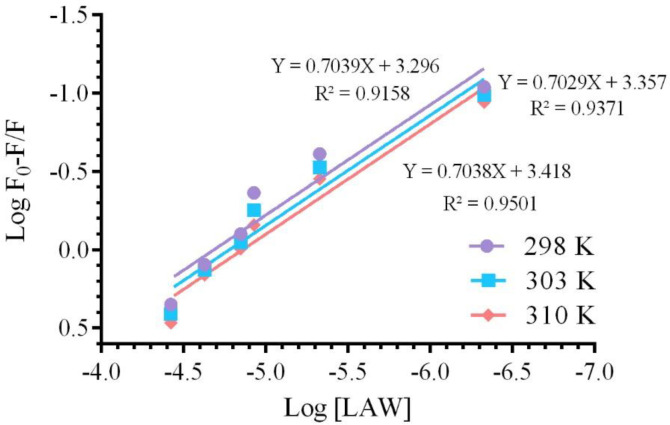
The linear plot of Log F0-F/F vs. Log [LAW] for quenching of BLC in the presence of LAW at 298, 303 and 310 K

The binding constant *K*_a_ and the number of binding sites *“n”*, were calculated by using the plot *vs. log [Q] *(Figure 7) and the results were listed in Table 1. 

*K*_a _and *“n”* were determined by using the intercept and slope values, respectively. The number of binding sites at both temperatures (25, 30 and 37 °C) was determined approximately equal to 1 (*n*≈ 1), describing that one molecule of LAW can bind to one molecule of catalase and CAT has only one binding site for LAW. The *K*_a _was enhanced by increasing the temperature, originated from an endothermic interaction between the CAT and LAW. The *K*_a _value was ≈10^3^, illustrating that there is a strong binding force between LAW and CAT(30). There is diverse mode of interaction between proteins and ligands such as electrostatic interactions, hydrogen bonds, hydrophobic forces, and van der Waals (11).Utilizing the thermodynamic parameters including enthalpy change (ΔH), entropy change (ΔS), and free energy change (ΔG), binding forces was estimated(31):1) ∆H ˂ 0 and ∆S > 0 imply that electrostatic forces is more important, 2) ∆H ˂ 0 and ∆S ˂ 0 illustrate hydrogen bonding to play the main role in the interaction, 3) ∆H ˃ 0 and ∆S ˃ 0 suggest hydrophobic bounding is dominant and 4) ∆H ˃ 0 and ∆S ˂ 0 indicate van der Waals forces are main (21, 32). van’t Hoff and the following equations (6 to 8) were used to calculate the changes in enthalpy (ΔH), Gibbs free energy(ΔG), and entropy (ΔS), respectively (10). All of thermodynamic parameters are given in Table 1.

$\ln\frac{K_{2}}{K_{1}}=\frac{-∆H}{R}(\frac{1}{T_{2}}-\frac{1}{T_{1}})$                    (6)

∆G=-RTlnK                    (7)

∆G=∆H-T∆S                    (8) 

The positive values, obtained from enthalpy changes (∆H) and the entropy changes (∆S) reflected that the hydrophobic boundings play a major role in the arrangement of the LAW-CAT complex and the negative quantity of ΔG illustrated that interaction of LAW with CAT is a spontaneous process (3, 21, 33).

*UV-vis spectroscopic studies*

**Figure 8:**
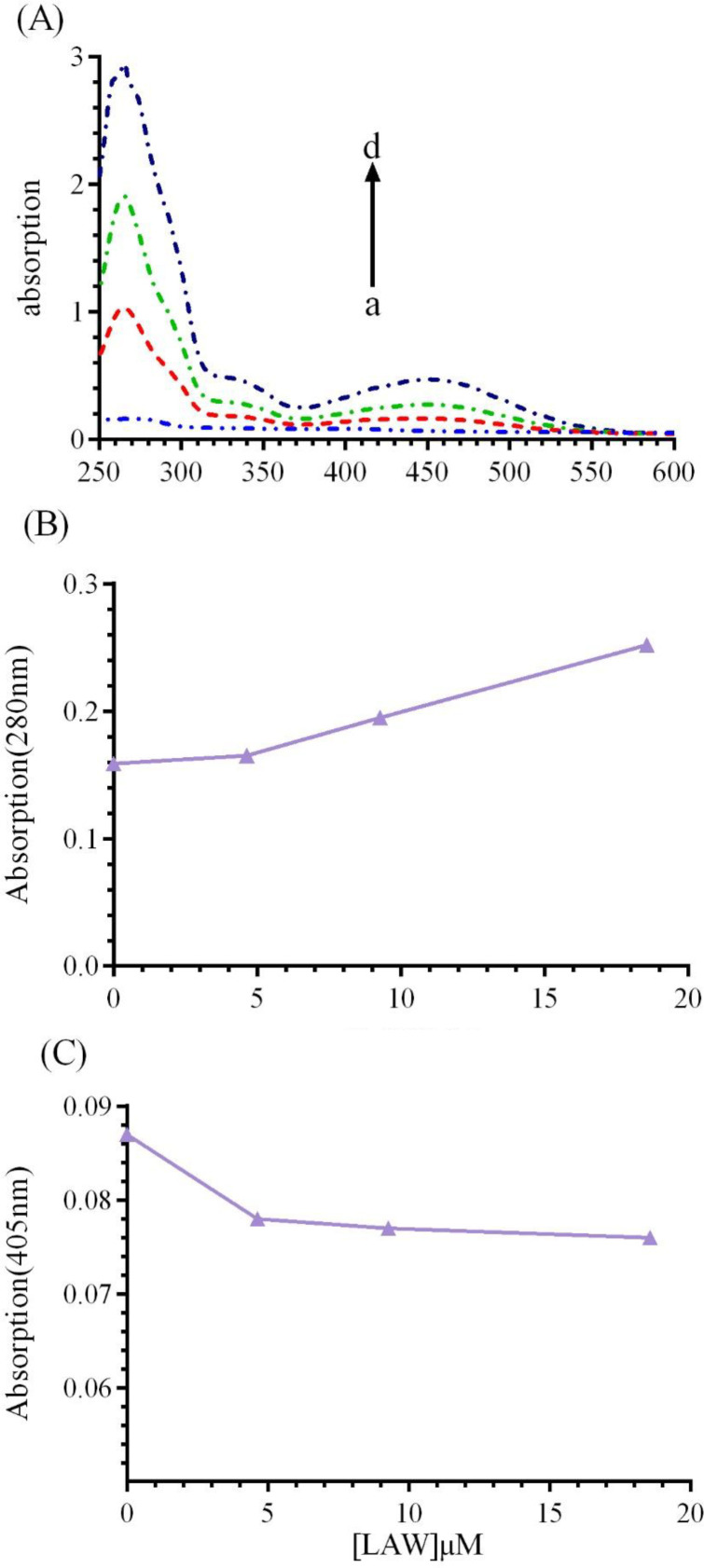
(A) UV-vis spectra of BCL (1μM) in the absence or presence of different concentrations of LAW; (a) 0, (b) 4.6, (c) 9.3 and (d) 18.6 µM in 50 mM phosphate buffer, pH 7, at 298 k, (B) LAW increases the absorption intensity of BLC at 280 nm, (C) LAW decreases the absorption intensity of BLC at 405 nm

UV-vis absorption study can be applied to explore the structural change of BLC upon interaction with LAW. BLC, like most of heme proteins has two principal absorption area: (1) an absorption band at 280 nm, which is resulted from the aromatic residues including tryptophan (Trp), phenylalanine (Phe), and tyrosine (Tyr) (8,30) and (2) Soret absorption band around 405 nm, which corresponding to π→π* transfer of electrons in the heme group(11).UV-vis spectra of CAT (1 μM) were monitored in the presence of growing concentrations of LAW (4.6, 9.3 and 18.6 µM) at 298 K (Figure 8). The results indicated that with raising the concentration of LAW, the maximum absorption intensities at 280 nm and 405 nm were enhanced and weakly decreased, respectively. Raising the absorbance at 280 nm shows that the interaction between CAT and LAW leads to change in Trp position and protein conformation. Weak change in the absorbance intensity at 405 nm illustrates a minor structural perturbation in the active site of the CAT.

*ATR-FTIR spectroscopy*

**Figure 9:**
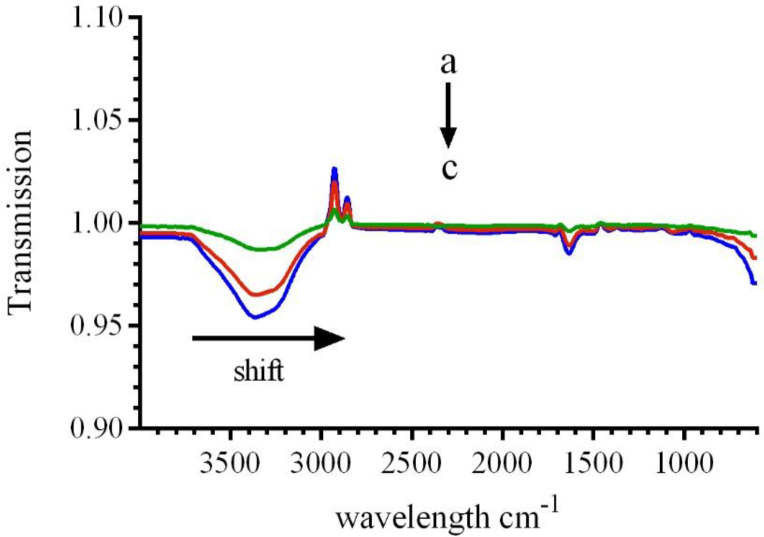
FTIR spectra of BLC (0.7 µM) in the absence or presence of different concentrations of LAW; (a) 0, (b) 9.4, (c) 23.6 in 50 mM phosphate buffer, pH 7, at 298 k

In order to prove the changes in the secondary structure of the CAT, FT-IR spectroscopic analyses were conducted. The FT-IR spectra of catalase demonstrated three amid bonds (I, II and III). Among the several binding groups of CAT, the amide I in the range of 1600–1700 cm^−1^(generally caused by C=O stretching vibration), is more sensitive to conformational variations and is mostly applied to analyze the secondary conformation of the proteins. The absorbance intensity of the amide II band in the range of 1500–1600 cm^−1^ (resulted from C-N stretch vibration combined with N-H bending mode) has been illustrated to be related to the amount of the enzyme-ligand complex. As shown in Figure 9, addition of various concentrations of LAW leads to change in FT-IR spectra of BLC. A shift in amide I from 1637.5 cm^−1^ (for free CAT) to 1635.5cm^−1^ and 1632.6 for additional concentrations of LAW (9.4 and 23.6 µM), respectively, and reducing the intensity demonstrated the change of the secondary structure and loss of CAT functionality. The amide II, which is located at 1537.2 cm^−1^had no position shifts, while the intensities were decreased significantly in the LAW-BLC complex, which indicated the reason of maintaining the certain activity of the enzyme. Previous studies have demonstrated that a shift in amide I bands is resulted from various secondary con formational elements: 1620–1645 cm^−1^from β-sheet, 1645–1652 cm^−1^for random coils, 1652–1662 cm^−1^from α-helix, and 1662– 1690 cm^−1^for turns. So LAW mainly changes the secondary structure of β-sheets in BLC structure (27, 34, 35).

*Molecular docking studies*

**Figure 10:**
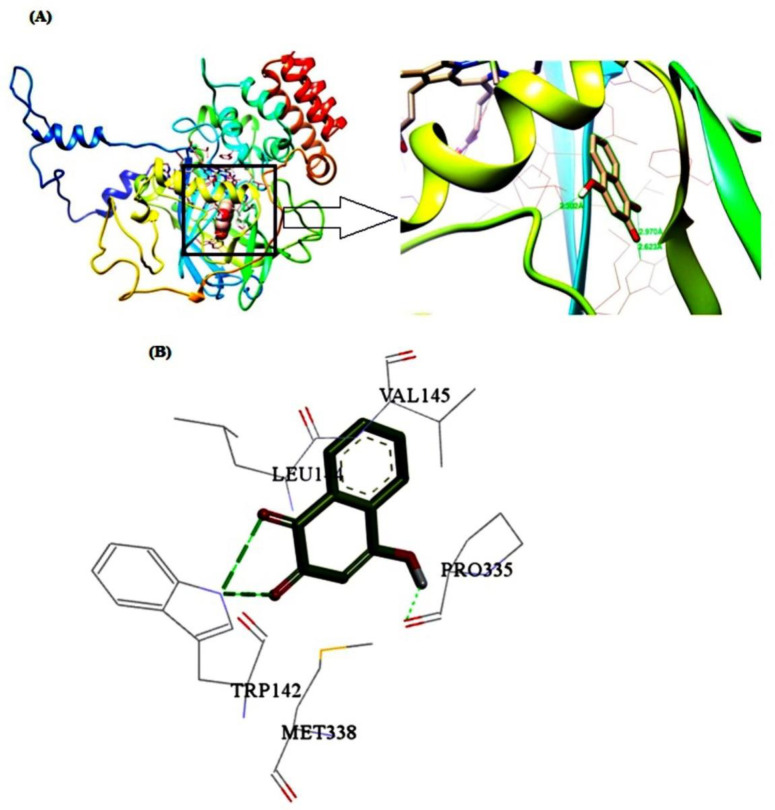
Molecular docking models for BLC-LAW complex. (A) The structure of one subunit of BLC binding site for LAW. (B) Detailed illustration of the binding between BLC and LAW

To identify the details of the best binding sites and dominant interaction of the ligand-macromolecule complex, the molecular docking computation was carried out based on the minimum energy. According to the obtained results, CAT has one binding site for LAW which is located close to the heme group and between the β-barrel, helical domain and wrapping domain of CAT (Figure 10). Five residues including Trp142, Leu144, Val145, Pro335, and Met338 are involved in the interaction of BLC and LAW. These results are in agreement with synchronous fluorescence data discussed earlier. The driving forces in the interaction between BLC and LAW are composed of hydrophobic and hydrogen bondings, whereas the prior boundings are dominant. The hydrogen bonds were formed between Trp142 and Leu144 as an electron acceptor and electron donor, respectively. The quantity of lowest binding free energy was computed as -3.85 kcal mol^1 ^(3, 12, 36). Different values of free energy, obtained from fluorescence and docking simulations may result from various factors such as rigidity of receptor, flexibility of ligand, and effect of buffer solution.

**Table 1.** Binding and thermodynamic parameters of BLC and LAW complex at different temperatures: (25 °C, 30 °C and 37 °C).

**T (K)**	**n**	**K**_SV_ ** (×10** ^4^ **M** ^-1^ **)**	**K (×10** ^3^ **M** ^-1^ **)**	**ΔG kJmol** ^-1^	**ΔHkJmol** ^-1^	ΔS JK^-1^mol^-1^
298	0.7	5.7	1.97	-18.79	10.23	98.08
303	0.7	6.4	2.2	-19.38		
310	0.7	7.2	2.6	-20.26		

## Conclusion

The present study investigated the effect of LAW, as an anti-oxidantand, anti-cancer and active component of Henna leaves, on the conformation and function of CAT by using various techniques including kinetic study, UV-vis absorption, fluorescence spectroscopy, FT-IR, synchronous fluorescence, and molecular docking approaches. This work indicated that LAW can bind to free enzyme or enzyme-substrate complex through one binding site and changes in the secondary structure and inhibits the activity of CAT by noncompetitive inhibition mechanism. The fluorescence spectroscopic data illustrated that LAW quenches fluorescence intensity of CAT through a dynamic quenching mechanism, and synchronous fluorescence calculations illustrated the alteration in the microenvironment of Tyr and Trp residues by LAW. UV-vis absorption studies revealed that the structure of CAT changes in the presence of LAW. ATR-FTIR spectroscopic data showed a shift in amide I bands, indicating alterations in the secondary structure of the enzyme. In order to determine the best binding condition between LAW and CAT, the molecular docking studies were performed. Our study may provide valuable information to figure out the importance of LAW in the treatment of the cancer cells through inhibition of catalase, due to high concentration of ROS such as H_2_O_2_that could be a therapeutic alternative for the treatment of cancer.
